# Leaderless foot-and-mouth disease virus serotype O did not cause clinical disease and failed to establish a persistent infection in cattle

**DOI:** 10.1080/22221751.2024.2348526

**Published:** 2024-04-29

**Authors:** Benedikt Litz, Julia Sehl-Ewert, Angele Breithaupt, Anja Landmesser, Florian Pfaff, Aurore Romey, Sandra Blaise-Boisseau, Martin Beer, Michael Eschbaumer

**Affiliations:** a Institute of Diagnostic Virology, Friedrich-Loeffler-Institut, Greifswald-Insel Riems, Germany; b Department of Experimental Animal Facilities and Biorisk Management, Friedrich-Loeffler-Institut, Greifswald-Insel Riems, Germany; c Animal Health Laboratory, Foot-and-Mouth Disease Reference Laboratory, Virology JRU, ANSES, INRAE, ENVA, Paris-Est University, Maisons-Alfort, France

**Keywords:** Foot-and-mouth disease virus, FMDV, persistence, carrier, leader proteinase

## Abstract

The foot-and-mouth disease virus (FMDV) Leader proteinase L^pro^ inhibits host mRNA translation and blocks the interferon response which promotes viral survival. L^pro^ is not required for viral replication in vitro but serotype A FMDV lacking L^pro^ has been shown to be attenuated in cattle and pigs. However, it is not known, whether leaderless viruses can cause persistent infection in vivo after simulated natural infection and whether the attenuated phenotype is the same in other serotypes. We have generated an FMDV O/FRA/1/2001 variant lacking most of the L^pro^ coding region (ΔLb). Cattle were inoculated intranasopharyngeally and observed for 35 days to determine if O FRA/1/2001 ΔLb is attenuated during the acute phase of infection and whether it can maintain a persistent infection in the upper respiratory tract. We found that although this leaderless virus can replicate in vitro in different cell lines, it is unable to establish an acute infection with vesicular lesions and viral shedding nor is it able to persistently infect bovine pharyngeal tissues.

## Introduction

The single-stranded (+)RNA foot-and-mouth disease virus (FMDV) belongs to the genus *Aphthovirus* of the family *Picornaviridae*. It causes vesicular lesions in the mouth, on the tongue, muzzle and in the interdigital cleft in cloven hoofed animals including pigs. The acute phase with its clinical lesions is accompanied by fever, shedding of high viral loads and a decline in productivity [1]. Vaccination and rigorous culling have successfully eradicated FMDV in Europe [2] but the reintroduction of FMDV into free countries poses a severe risk and causes dramatic economic losses as seen in the United Kingdom in 2001 [3]. The acute phase of FMDV infection is followed by a persistent phase in more than 50% of infected cattle, the so-called carriers [4–7], whereas pigs do not become persistently infected [8]. The persistent infection is characterized by an absence of clinical lesions but infectious virus can be recovered from the animal after the 28th day of infection [9]. In contrast to a generalized subclinical infection, the persistent infection in cattle is strictly limited to epithelia of the upper respiratory tract, mainly the dorsal nasopharynx and the dorsal soft palate [10]. Microanatomically, the follicle-associated epithelium (FAE) overlying the mucosa-associated lymphoid tissue (MALT) has been described as the preferential location [4]. As this site is not easily accessible for sampling, special probang cups are used for the collection of oropharyngeal fluid (OPF) from the oesophagus. Although live virus can be recovered from persistently infected animals, the contagiosity of persistently infected cattle is still debated as no convincing evidence for onward transmission has been found [8,11]. However, in the natural host for FMDV, the African buffalo (*Syncerus caffer* [Sparrman, 1779]), persistently infected animals have been observed to transmit virus to naïve buffalo and cattle under experimental conditions [12,13]. A transmission model informed by the experimental data suggested that carrier buffalo are important for the maintenance of FMDV endemicity in buffalo populations [12].

The single open reading frame (ORF) of the FMDV genome encodes 4 structural proteins and 10 non-structural proteins, of which the Leader proteinase L^pro^ is the first to be translated and forms the N-terminus of the polyprotein. The coding sequence of L^pro^ contains two start codons as alternative translation initiation sites, termed Lab and Lb, 84 nucleotides apart [14]. A functional difference between the two proteins has not been observed [15]. The deletion of the L^pro^ coding region after the second start codon Lb allows the rescue of viable virus, but complete deletion of the entire L^pro^ does not. The inter-AUG region between the start codons has therefore been demonstrated to be necessary for viral replication. The Lab start codon can be mutated as long as the inter-AUG region and the Lb start codon remain intact, whereas the mutation of the Lb start codon does not produce viable virus [16].

Post-translational release of the papain-like protease L^pro^ from the structural protein VP4 in the FMDV polyprotein takes place by self-cleavage [17]. An important function of L^pro^ is the inhibition of the host cell translation by cleaving the initiation factor eIF4G [18], resulting in the shutdown of the cap-dependent mRNA translation. Translation of the viral RNA of FMDV is not affected due to the internal ribosome entry site (IRES) in the 5'UTR which mimics the cellular translation initiation complex by its three-dimensional RNA secondary structure. In addition to an enhancing viral replication at the expense of the host, L^pro^ inhibits the interferon (IFN) response by either cleaving or deubiquitinating important transcriptional factors such as IRF3, IRF7, TRAF3 or RIG-I, as has been demonstrated *in vitro* [19,20]. *In vivo*, this may be less effective, as strong viral replication tends to induce rather than suppress the interferon response [21].

Some FMDV variants have been constructed that lack the Leader proteinase. Piccone et al. [22] produced a variant based on FMDV A_12_, by removing the L^pro^ coding sequence following the second start codon Lb. It grew more slowly in BHK-21 cells and was slightly attenuated in suckling mice compared to wildtype FMDV. After aerosol inoculation of cattle with the A_12_ mutant, the animals showed no clinical signs at 72 h post infection (hpi) and only focal virus replication, primarily in the lung [23]. Following this initial *in vivo* characterization, a further challenge experiment was carried out using intradermal or intramuscular inoculation of the leaderless A_12_, which induced protective immunity against a homologous challenge. Oropharyngeal fluid was collected in this animal study, but no virus could be isolated prior to challenge infection at 35 days post infection (dpi) [24]. Uddowla et al. [25] used FMDV A_24_ Cruzeiro with the same deletion to inject animals intradermolingually. No clinical signs or viral shedding were observed but the inoculated cattle did develop neutralizing antibodies. In the same study, animals were vaccinated with a chemically inactivated leaderless A_24_ Cruzeiro formulated with adjuvant and were protected from clinical FMD after homologous challenge.

The O1K ΔLb mutant constructed by Belsham et al. has the same deletion of the Lb coding sequence in the backbone of FMDV O_1_ Kaufbeuren and showed similar growth kinetics in BHK-21 cells as wildtype O1 K, but it was unable to infect primary bovine thyroid cells [16]. Another method that achieved attenuation in cattle with unimpeded replication *in vitro* was a 54-nt in-frame insertion in the inter-AUG region, maintaining the functionality of L^pro^ [26].

When evaluating the safety of leaderless viruses, recombination of a leaderless virus with a Leader proteinase from a closely related virus such as bovine rhinitis B virus (BRBV), which is ubiquitous in cattle [27], should be considered. Picornaviruses having a high recombination rate [28], with breakpoints for recombination in the FMDV genome following L^pro^ [29]. Uddowla et al. have addressed this issue by showing that such a chimeric virus is also fully attenuated in cattle and of low virulence in swine [30].

Long-term persistence of leaderless virus in infected animals could constitute a reservoir for potential recombination. The aim of this study was therefore to create an FMDV variant from a more recent virulent FMDV isolate (O/FRA/1/2001), to characterize its early infection dynamics after simulated natural infection via the intranasopharyngeal route and to determine whether it is capable of persistently infecting cattle and remaining in the epithelia of the upper nasopharynx.

## Material and methods

### Generation of leaderless FMDV

A plaque-purified isolate of FMDV O/FRA/1/2001 was provided by the Animal Health Laboratory, ANSES, Maisons-Alfort, France, and a cow was experimentally infected with this clone at the BSL4vet facility of the FLI in Riems. Vesicular material from this cow was sequenced on an Ion Torrent platform as previously described [31] (Genbank accession no. OV121130.1). The entire genome including both UTRs (with a 13-mer poly-C region in the 5'UTR) was synthesized by GeneArt (Regensburg, Germany). The cDNA was inserted into the original pT7S3 plasmid [32] by restriction-free cloning [33], replacing the entire O1 K genome. Clones with the correct insertion were selected after a restriction enzyme digestion and the insertion was verified by Sanger sequencing using the universal FMDV primer set of Dill et al. [34]. Confirmed clones were linearized by *Hpa*I digestion and transfected into BSR-T7 cells, a BHK-21 cell line expressing T7 RNA polymerase [35], using 1 µl of Lipofectamine 3000 and 500 ng of linearized DNA in a 12-well plate.

The deletion of the Lb coding sequence from the O/FRA/1/2001 infectious clone was performed with the Q5 site-directed mutagenesis kit (New England BioLabs) using primers 5'-GGCGCCGGGCAATCCAGC-3' and 5'-CATCTTTCCTTGTGCTCGTGATAAGAACAGTGTTTTAATCTC-3'. Transformation, selection and sequencing of correct clones were carried out as described above. For transfection 1.5 µl lipofectamine was used with 500 ng of linearized DNA and the cell culture plates were centrifuged for 1 h at 800×*g* after transfection to increase efficiency [36].

### Growth kinetics

Viral growth kinetics were examined in a comparison between the parental strain O/FRA/1/2001 wildtype (WT) and its leaderless derivative O/FRA/1/2001 ΔLb at a multiplicity of infection (MOI) of 0.1 on BHK-21 cells (CCLV-RIE 0164, Collection of Cell Lines in Veterinary Medicine, FLI, Greifswald-Insel Riems, Germany), porcine kidney cells expressing bovine αVβ6 integrin (LFBK-αVβ6, CCLV-RIE 1419) [37], IB-RS-2 porcine kidney cells (CCLV-RIE 103) [38] and ZZ-R goat tongue cells (CCLV-RIE 127) [39]. Samples were taken on time points 0, 4, 8, 16, 24 and 42 hpi and titrated on BHK-21 cells. The data were collected from three biological replicates.

### Animal trial

The animal experiment was carried out under BSL4vet conditions at the Riems site of the FLI. Sixteen Holstein–Friesian heifers of around 4 months of age and with an average body weight of 110 kg were obtained from the same breeder and randomly assigned to two groups of eight animals. The groups were housed in separate rooms and observers were not blinded to treatment group assignment. After 1 week of acclimatization to the containment facility, the animals were inoculated via intranasopharyngeal instillation, which closely simulates natural infection [40]. One group was inoculated with O/FRA/1/2001 ΔLb and the other was inoculated with the recombinantly produced parental strain O/FRA/1/2001 WT. Both viruses had been passaged twice on BHK-21 cells. The cultures were frozen and thawed and the supernatants were clarified by centrifugation. Virus titres were determined by end-point titration on BHK-21 cells. For inoculation, the virus preparations were diluted in cell culture medium (minimum essential medium with Hanks’ and Earle's salts and non-essential amino acids) to adjust the virus concentration and applied in a single dose of 0.8 × 10^7^ TCID_50_ in 2 ml medium. We used the highest dose possible based on the available volumes and titres of the virus preparations to demonstrate the safety of the leaderless virus even when applied at a high dose.

For inoculation as well as for clinical examinations on days 2, 4, 6, 8 and 10 pi, the animals were sedated with 0.3 mg/kg xylazine. The sedation was reversed by atipamezole at a dose of 0.025 mg/kg. Rectal body temperatures were recorded daily. To avoid the potentially confounding use of anti-inflammatory drugs for analgetic treatment [6], buprenorphine at 0.01 mg/kg was given daily to animals with vesicular lesions during the acute phase. Animals which showed signs of bronchopneumonia were treated with 750 mg of enrofloxacin per animal.

Out of each group, two animals were euthanized 24 hpi, the remaining animals were euthanized on day 35 or 36 pi. For euthanasia, animals were lightly sedated with xylazine at 0.05 mg/kg and led to the necropsy room. There they were deeply sedated with xylazine at 0.3 mg/kg and euthanized by intravenous administration of 90 mg/kg pentobarbital. Once pain reflexes had ceased, the animals were exsanguinated.

The protocol for the animal trial (file no. 7221.3-1-052/21) has been approved by the State Office for Agriculture, Food and Fisheries of Mecklenburg-Vorpommern (LALLF M-V).

### Ante and post mortem sample collection

As a negative control, serum, nasal fluid, saliva and OPF were collected from each animal before infection. Immediately after inoculation, an additional sample of nasal fluid was collected. From 1 to 10 dpi, serum, nasal fluid and saliva were collected each day, thereafter samples were taken on days 14, 17, 21, 24, 28, and 31 pi. The collection of OPF with a special probang cup was started on 7 dpi in the ΔLb group. In the WT group, the first OPF sample was taken on 10 dpi to avoid contamination by recently ruptured vesicles in the oral cavity. The collection of OPF was carried out on the same days as listed above with an additional sampling on 35/36 dpi before euthanasia. During necropsy, a set of tissue samples was collected representing different epithelial surfaces in the pharynx, the lungs, and their draining lymph nodes (see Table 1). Tissue samples from the lungs were only collected from animals euthanized 24 hpi. From each tissue, one specimen was archived for histopathological examination, while the remainder was used for genome detection and virus isolation. Tissues from the dorsal soft palate (DSP) and the dorsal nasopharynx (DNP) were split into five biological replicates per region to address the focal nature of persistent infection. Tissue samples for RT-qPCR and virus isolation were frozen over liquid nitrogen immediately after collection and then stored at −80°C.

**Table 1.** Summary of tissue samples collected at necropsies 24 hpi and 35/36 dpi.

	24 hpi	35 dpi
Ventral soft palate (VSP)	X	X
Dorsal soft palate (DSP)	X	X
Dorsal nasopharynx (DNP)	X	X
Pharyngeal tonsil	X	X
Laryngeal epithelium at the base of epiglottis	X	X
Lung, right proximal cranial lobe	X	
Lung, right proximal distal lobe	X	
Lung, right medial lobe	X	
Lung, right caudal lobe	X	
Retropharyngeal lymph node	X	X
Submandibular lymph node	X	X
Tracheobronchial lymph node	X	

### RNA extraction from samples and tissues

RNA was extracted from 100 µl of serum, nasal fluid, saliva and OPF using the NucleoMag Vet kit (Macherey-Nagel) with a King Fisher Flex (Thermo Scientific) magnetic particle processor. As an internal control, 10 µl of IC2 RNA were added during the extraction [41]. Tissue samples collected during necropsy were disintegrated in 750 µl of PBS using a 5-mm steel ball in a TissueLyser II (Qiagen) for 2 minutes at 30 Hz. Supernatants were collected after centrifugation and used for RNA extraction and virus isolation.

### Genome detection

FMDV genome was detected and quantified by RT-qPCR using AgPath–ID One–Step RT–PCR reagents (Thermo Fisher Scientific) with a primer/probe set targeting the 3D-coding region [42]. To detect concurrent infection with BRBV, a possible donor for the reacquisition of a leader protease through recombination, all animals were tested by RT-qPCR using primers and a probe published by Xie et al. [43].

### Virus isolation from collected samples

Sampling with the probang cup was performed to determine if animals were persistently infected [44]. Recovered OPF was mixed with 4 ml of cell culture medium. The liquid was then homogenized by repeated aspiration with a 16G blunt cannula before half of the sample was mixed with an equal amount of 1,1,2-trichloro-1,2,2-trifluoroethane (TTE) [45]. This mixture was vigorously shaken for 5 min and afterwards centrifuged at 1000×*g* for 10 min at 4°C. The supernatant was removed and aliquoted. A 90% confluent LFBK-αVβ6 monolayer in 25 cm^2^ culture flasks was inoculated with 250 µl of the TTE-treated OPF. For the ΔLb group, this was repeated with BHK-21 cells. Positive virus isolation (i.e. cytopathic effect) was confirmed by FMDV RT-qPCR as described above. Two passages were performed to confirm negative results.

For virus isolation from tissues, the supernatants were mixed with an equal amount of TTE and then vigorously shaken for 5 min, afterwards the mixture was centrifuged at 900×*g* for 20 min at 4°C [46]. A volume of 50 µl supernatant were used for virus isolation on LFBK-αVβ6 cells. Only samples with a positive FMDV RT-qPCR result were selected for virus isolation.

### Serology

Antibodies against non-structural proteins (NSP) of FMDV were detected with the PrioCHECK FMDV NS ELISA (Thermo Fisher Scientific) using the overnight protocol.

### Histopathology

Collected tissue samples (see Table 1) were fixed in 10% neutral-buffered formalin for at least 3 weeks and processed for paraffin-embedding. Embedded tissues were cut at 2–3 µm thick sections, mounted on glass slides, dewaxed in xylene, and rehydrated in descending graded alcohols. For morphological evaluation, sections were stained with haematoxylin–eosin (HE) following standard procedures.

To detect viral RNA, RNA *in situ* hybridization was performed on selected tissues (DSP, DNP) obtained from animals which were tested virus positive at the end of the experiment. RNAScope probes were custom-designed against the highly conserved FMDV NSP 3D (ACD, Advanced Cell Diagnostics, Newark, CA, USA) and used with the corresponding RNAScope 2–5 HD Reagent Kit-Red according to manufacturer`s instructions. As technical assay controls, a positive control probe for the housekeeping gene peptidylprolyl isomerase B (cyclophilin B, *PPIB*) and a negative control probe for dihydrodipicolinate reductase (*DapB*) were included.

### Statistical analysis

The binomial proportion confidence interval for the incidence of persistent FMDV infection in the wildtype group was calculated by the Wilson method [47] using R (https://www.r-project.org/).

## Results

### Virus replication *in vitro*

An infectious clone of FMDV O/FRA/1/2001 was created by inserting commercially synthesized cDNA in the pT7S3 plasmid. Cytopathic effect (CPE) was observed after the transfection of BSR-T7 cells with the plasmid containing the O/FRA/1/2001 WT sequence. After two passages on BHK-21 cells, the titre of the recombinantly produced WT virus was 1.21 × 10^7^ TCID_50_/ml.

Its leaderless derivative O/FRA/1/2001 ΔLb was also passaged twice on BHK-21 cells and then titrated on the same cell line. Its titre was 1.00 × 10^7^ TCID_50_/ml.

**Figure 1.**
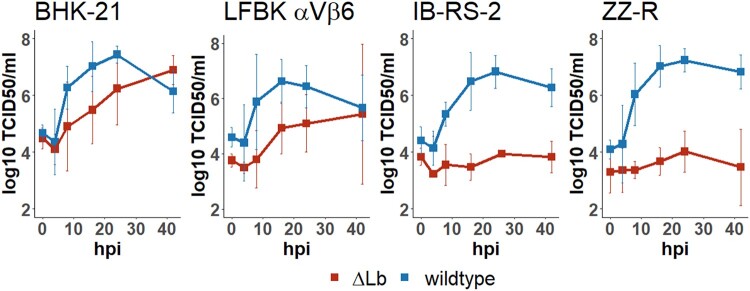
*In vitro* replication of O/FRA/1/2001 wildtype versus leaderless (ΔLb) FMDV over 42 h on four cell lines (BHK-21, LFBK αVβ6, IB-RS-2, and ZZ-R) infected at an MOI of 0.1. Supernatant was collected at 0, 4, 8, 16, 24, and 42 hpi and titrated on BHK-21 cells.

In BHK-21 cells, growth kinetics and final titre were similar between the leaderless mutant O/FRA/1/2001 ΔLb and the parental virus as shown in Figure 1. In LFBK-αVβ6 cells, growth of the mutant was delayed and did not reach titres as high as the wildtype. In IB-RS-2 as well as in ZZ-R cells, on the other hand, the mutant was strongly inhibited in its growth.

### *In vivo* attenuation of leaderless FMDV without virus shedding

In the animal trial, two groups of eight cattle were inoculated with WT FMDV O/FRA/1/2001 or its leaderless derivative and two animals of each group were euthanized 24 hpi. Clinical signs consistent with FMD were only observed in the animals of the WT group. All remaining animals in this group developed vesicular lesions beginning on 4 dpi. Prominent vesicular lesions were seen on the tongue, dental plate, gingiva, muzzle and in the nostrils. Five of six animals also had lesions on all four extremities; animal 770 only had lesions in three of four interdigital clefts on 14 dpi. No lameness or recumbency was observed in any of the heifers.

ΔLb infected cattle did not develop any vesicular lesions or any other clinical signs of FMD.

**Figure 2.**
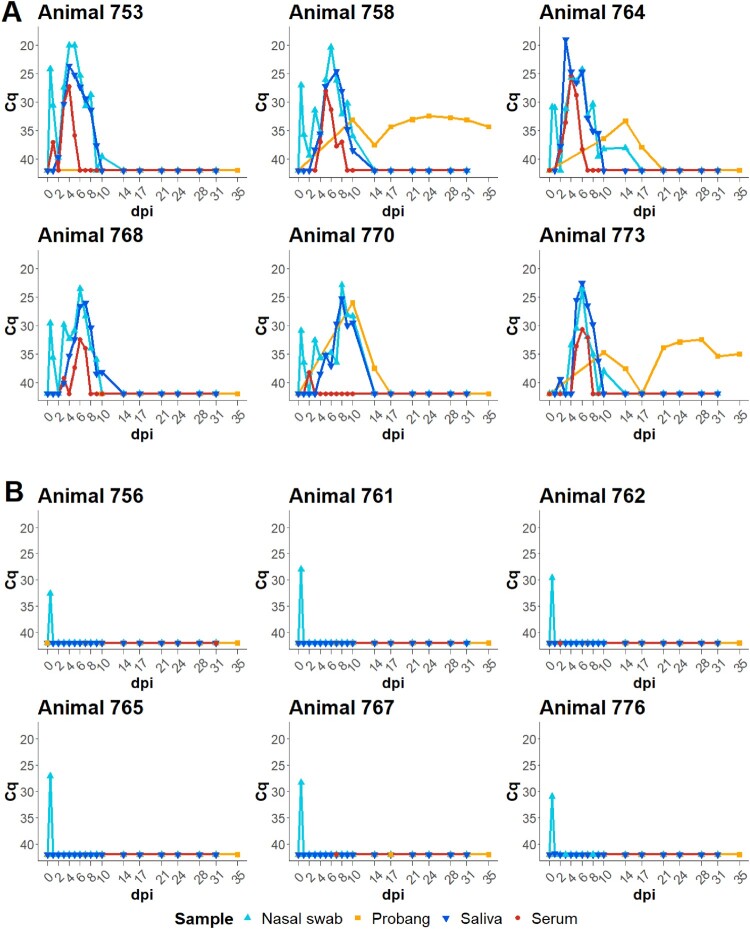
Ante-mortem infection dynamics of cattle infected with (A) wildtype FMDV or (B) FMDV ΔLb. The *C*_q_ values of the FMDV RT-qPCR for samples collected during the animal trial, including serum, nasal fluid, saliva, and OPF (probang) are shown on a reversed *y*-axis over the course of the experiment, from immediately before and immediately after inoculation on 0 dpi until the end of the trial on 35 dpi.

Figure 2 shows the results of the FMDV RT-qPCR for the WT (Figure 2A) and the ΔLb group (Figure 2B), respectively. All animals were positive for FMDV RNA in the nasal fluid sample taken immediately after intranasopharyngeal instillation of the WT or ΔLb virus, confirming the successful inoculation. In the ΔLb group, no viral RNA was detected in any other sample over the course of the experiment.

In the WT group, FMDV RNA detection in nasal fluid, saliva and serum peaked between 4 and 6 dpi. Viraemia was cleared in all animals before 10 dpi with animal 770 having detectable viraemia only at 2 dpi.

### Incidence of persistently infected carrier animals

According to the World Organisation for Animal Health, an animal is considered persistently infected with FMDV when virus can be recovered later than 28 days after infection [48]. This threshold is arbitrarily chosen and FMDV carriers can be already diagnosed at earlier time points [8]. However, we decided to use the standard definition for this study. TTE-treated OPF samples from two animals (758 and 773) in the WT group were consistently positive in the virus isolation from 10 dpi to the end of the experiment, resulting in an incidence of persistent infection of 33% (95% confidence interval 10–70%). In two other animals, 764 and 770, virus isolation was positive on 10 dpi only.

No virus was isolated from TTE-treated OPF from any animal in the ΔLb group on either LFBK-αVβ6 or BHK-21 cells, corresponding to an incidence of persistent infection of 0% (95% confidence interval 0–39%).

### Leaderless FMDV does not induce an antibody response

In the WT group, NSP antibody levels started to rise around day nine after infection, except for animal 770 which had a detectable antibody response only after 17 dpi. In the ΔLb group, no animal developed detectable anti-NSP antibodies over the course of the experiment (not shown).

### Viral RNA in tissues at 24 hpi versus 35 dpi

Of each infected group, two animals were euthanized 24 hpi, while the remaining six animals were euthanized on 35 dpi. From each animal, a set of tissue samples was collected at necropsy and tested for FMDV RNA by RT-qPCR. All tissue samples with a positive FMDV RT-qPCR were used for virus isolation. The sample set included epithelia of the pharynx and the draining lymph nodes. In the animals euthanized 24 hpi, the tracheobronchial lymph node and four samples from different regions of the lung were collected in addition.

In the two animals from the WT group that were euthanized at 24 hpi, FMDV RNA was detected in several tissues, with the highest viral load in the distal mid lobe of the lung followed by the pharyngeal tonsil as shown in Supplemental Table S1.

**Figure 3.**
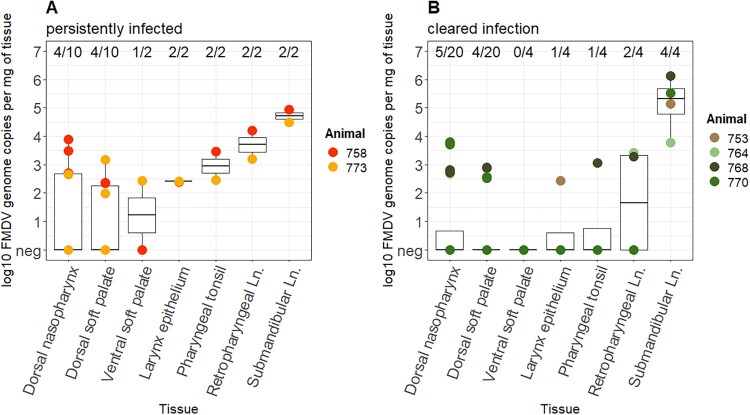
Tissue distribution of FMDV RNA in samples collected at necropsy on 36 dpi, compared between the two persistently infected animals 758 and 773 (i.e. animals from whose OPF virus was isolated after 28 dpi) (A) and the four animals 753, 764, 768, and 770 that had cleared the infection (i.e. had no detectable virus in OPF) at the time of euthanasia (B). The following tissues were collected from each animal: dorsal nasopharynx (*n* = 5), dorsal soft palate (*n* = 5), ventral soft palate (*n* = 1), larynx epithelium (*n* = 1), pharyngeal tonsil (*n* = 1), retropharyngeal lymph node (*n* = 1), and submandibular lymph node (*n* = 1). Their FMDV RNA content was quantified by RT-qPCR and is presented as log_10_ genome copy numbers per mg of tissue. The boxplots represent the distribution of FMDV content in each tissue including negative samples. The proportion of positive samples for each tissue is indicated by the fraction at the upper edge of the panels. Neg, negative; Ln, lymph node.

At the end of the experiment, several tissues of WT cattle were positive in the FMDV RT-qPCR as depicted in Figure 3. Irrespective of the animal's carrier status, the highest viral genome loads in animals euthanized on day 35 or 36 pi were detected in the lymph nodes, particularly in the submandibular lymph node, followed by the retropharyngeal lymph node and the samples taken from the dorsal nasopharynx. Several epithelial tissues as well as the majority of lymph nodes of animals in the WT group contained detectable FMDV RNA. Overall, the four animals that successfully cleared the infection had similar amounts of FMDV RNA in the same tissues as the two carrier animals, but a lower proportion of positive samples overall (17/60 or 28% vs. 17/30 or 57%, as shown in Figure 3).

Virus isolation with tissue samples from 36 dpi was only successful in one (animal ID: 758) of the two carrier animals, whose carrier status had been previously defined by the virus isolation from OPF. The positive tissue samples from this animal were DNP (*n* = 2), DSP and larynx (both *n* = 1) as described in Supplemental Table S1. Despite the high viral genome loads in the sampled lymph nodes of WT-infected animals, it was not possible to recover live virus from these tissues.

No tissue collected at 24 hpi or 35 dpi from animals infected with ΔLb contained any detectable FMDV RNA.

### Localization of viral RNA in tissues of persistently infected animals

**Figure 4.**
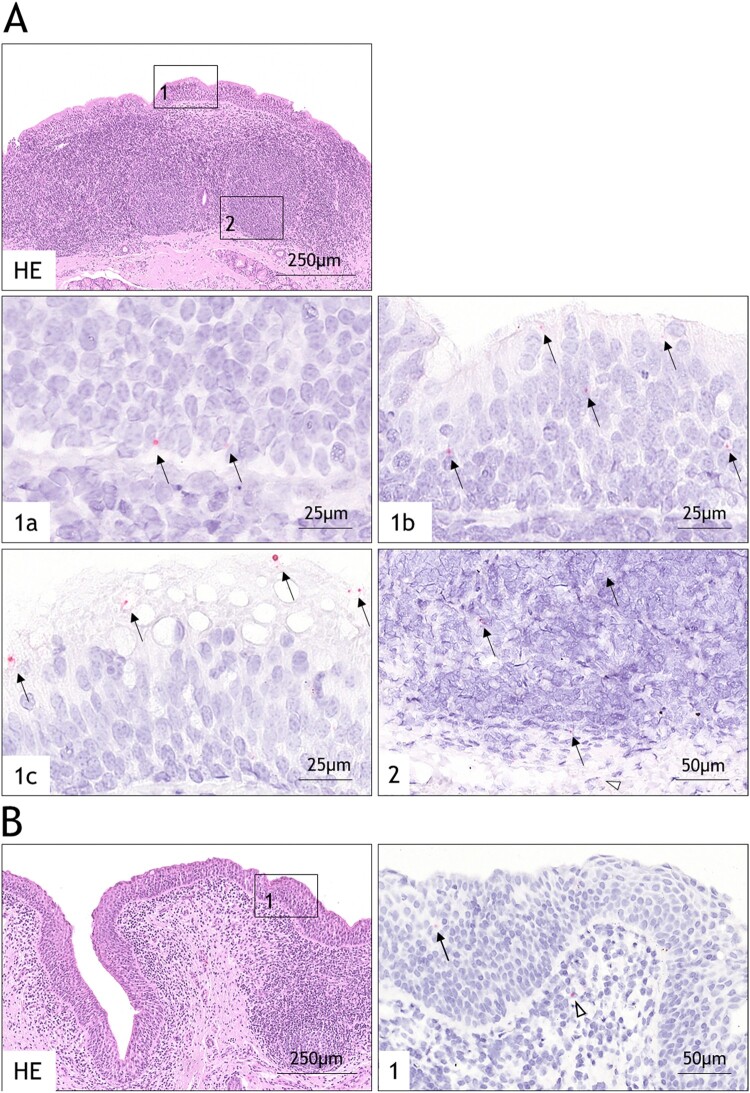
Histopathological findings using RNA *in situ* hybridization for tissues of carrier animal 758 infected with wildtype FMDV strain O/FRA/1/2001. HE stains are shown for morphological orientation in the tissues only. Consecutive sections were used for the *in situ* hybridization. (A) HE-stained overview of the dorsal soft palate (DSP) showing the approximate location of positive FMDV NSP 3D-specific RNA signals in the ciliated pseudostratified columnar epithelium (box 1) and submucosal lymphoid tissue (box 2). On consecutive sections, arrows indicate viral RNA within basal cells (1a), within columnar cells (1b), on the apical surface of columnar cells (1c) and within submucosal lymphoid follicles (2), stained by *in situ* hybridization. (B) Overview of the dorsal nasopharynx (DNP) indicating the approximate location of positive RNA signals (box 1), HE stain. On consecutive sections, compared to the DSP, viral RNA was found in fewer cells in the epithelium (arrow) and submucosa (arrowhead) by *in situ* hybridization.

On the basis of positive virus isolation at 36 dpi, the DSP and DNP of the two carrier animals (animal IDs: 758 and 773) were investigated with RNA *in situ* hybridization. While in animal 758 NSP 3D RNA was detectable in both tissue samples, in 773 only the DSP revealed positive signals (for comparison of staining in acutely infected tissue, see Figure S1). Viral RNA was mainly found in the ciliated pseudostratified columnar epithelium and less often in the submucosal lymphoid follicles and stromal cells of the DSP. Positive signals were identified throughout the epithelium within basal cells as well as within and on the apical surface of columnar epithelial cells (Figure 4A). In contrast, only single epithelial and submucosal stromal cells of the DNP were positive for viral RNA (Figure 4B).

## Discussion

Any work with FMDV has to be carried out under the highest biosecurity conditions. Even though containment restrictions have become very sophisticated, vaccine production involving large amounts of infectious virus carries a significant risk of inadvertent release of FMDV. Producing inactivated vaccines using a strongly attenuated virus with the same capsid proteins offers a considerable safety advantage. *In vivo* attenuation of leaderless FMD viruses was successful with serotypes O and A [16,22], while protection afforded by inactivated vaccines prepared from a leaderless virus has been described only for serotype A [25,49]. Mason et al. [24] also evaluated the carrier status of animals inoculated with leaderless FMDV O but did not find any. However, in their study the leaderless virus had been injected subcutaneously, which is a highly artificial route of delivery for live FMDV and may not be able to cause persistent infections at all.

For our animal trial, the intranasopharyngeal instillation method was used, simulating the natural route of infection in the best possible way without requiring additional animals for contact exposure [40]. Contact exposure was not considered appropriate due to the expected strong attenuation of leaderless viruses. Our results support the previous finding of a strong attenuation of leaderless FMDV *in vivo* and we did not find any evidence that leaderless FMDV can persist in epithelia of the upper nasopharynx.

We compared an FMDV mutant lacking the Leader protease L^pro^ to its parental wildtype strain, genetically identical to a FMDV field isolate from the 2001 outbreak in France, but rescued by reverse genetics.

Both viruses were characterized *in vitro* by growth kinetics in four different cell lines. The similar growth of the leaderless ΔLb FMDV in BHK-21 cells is most likely due to the interferon deficiency of this cell line [50], removing any advantage given to the wildtype virus by the inhibition of the interferon response by L^pro^. Similarly, FMDV ΔLb grew well in our LFBK-αVβ6 cells, which are contaminated with a non-cytopathic strain of bovine viral diarrhoea virus (BVDV) known to inhibit the interferon response [51,52]. However, a comparison of the leaderless FMDV and its parental strain on BVDV-free LFBK-αVβ6 cells [42] would be interesting. In interferon-competent cell lines (IB-RS-2 and ZZ-R), leaderless FMDV grew to much lower titre than the wildtype. This is in line with previous reports about leaderless FMDV in primary cells [16]. Even in an air–liquid interface model of bovine dorsal soft palate, as a highly susceptible tissue *in vivo*, our O/FRA/1/2001 ΔLb strain was unable to replicate (Michaud et al., manuscript in preparation).

*In vivo*, the wildtype FMDV O/FRA/1/2001 caused clinical FMD. Every exposed animal became infected, developed vesicular lesions on hairless epithelia and shed virus in nasal fluid and saliva for up to 10 days. Viraemia lasted for several days, reaching its peak around days 4–6 post infection, except in animal 768, which was viraemic for only one day with a low viral genome load.

Of six animals in our study that remained in the experiment until day 35, two (animal IDs: 758 and 773) could be defined as carrier animals with several positive virus isolations from TTE-treated OPF after the 28th day of infection which was confirmed by the presence of FMDV NSP 3D-specific RNA in selected tissues. Due to the small number of animals, the confidence interval for the incidence of persistent infection in our study (9.7–70.0%) overlaps with findings of earlier studies, which reported varying incidences around 50% due to small animal numbers [8]. In detail, for serotype A the carrier incidence ranged from 62% to 94% [4–6,53], while for serotype O incidences between 25% and 100% have been documented using smaller animal numbers than for serotype A [7,54–56]. Two other animals with relatively high viral loads in OPF after the acute phase (764 and 768) may be of interest for identifying factors which lead to the clearance of persistent FMDV.

Overall, the recombinant O/FRA/1/2001 wildtype virus was highly infectious and caused clinical signs comparable to the original outbreak strain. In contrast to this, the leaderless derivative O/FRA/1/2001 ΔLb was neither able to cause acute disease nor did it persist in tissues of the nasopharynx. Although viral RNA was present in nasal fluid sampled right after the inoculation from every animal, none was detected later on, neither in body fluids nor in tissue samples. The absence of seroconversion in this group also supports the conclusion that no infection has occurred in the ΔLb group.

Our examination of the tissue distribution of wildtype FMDV in animals euthanized 24 hpi showed high viral genome loads in lung tissue, as reported by Brown et al. [23] who unlike us have used aerosol inoculation. The prominent replication of wildtype FMDV in the region of the nasopharynx in one heifer after 24 h was not observed by Brown et al. [23]. This differing tissue distribution could indicate a substantial difference between the inoculation methods used herein.

In samples taken at the end of the experiment, high amounts of viral RNA were detected in the lymph nodes of animals infected with the wildtype virus, especially in the submandibular lymph node. This is in accordance with the findings of Juleff et al. [57]. Despite the high viral genome loads, it was not possible to recover live virus from these tissues, which may be explained by only RNA remaining in the lymph nodes after clearance of virus by the cellular immune response [4]. The high viral genome loads found especially in the submandibular lymph node may be due to drainage from mucosa of the rostral skull, e.g. on the muzzle, nostrils, gingiva, and tongue [58], which are the main localization for vesicular lesions. The high viral RNA loads in lymph nodes in infected animals may be a result of clinical disease during the acute phase rather than the persistent infection.

The absence of detectable viral RNA in the lymph nodes of the animals in the ΔLb group, on the other hand, suggests that there was virtually no replication of this virus in the nasopharynx at any time. As seen by the high loads in submandibular lymph nodes of animals in the wildtype group, viral RNA remains detectable for 5 weeks after acute infection.

Viral RNA was detectable in different tissues of all WT-infected animals at the end of the trial, and we were able to recover virus from one carrier animal (758). The localization of the viral RNA was consistent with previous immunohistochemical studies of persistent FMDV infection. Immunofluorescence targeting structural protein VP1 found it mostly in the superficial layer of the epithelium [4,59,60]. Pacheco et al. [10] detected non-structural protein 3D preferentially in the basal layer and viral RNA was stained by *in situ* hybridization in the basal layer as well [61–63]. The detection of viral RNA in basal and columnar cells of the dorsal soft palate in the present study could indicate that, similar to papillomaviruses [64], FMDV targets the basal cell layer for initial persistent infection. In the basal cells, the viral genome is maintained in the absence of a productive viral lifecycle. Structural proteins and viral progeny are only produced upon differentiation of the epithelial cells into the more superficial columnar cells.

In at least one animal in our trial, the closely related BRBV was detected at the time of infection with leaderless FMDV. This is likely due to the generally high prevalence of BRBV in cattle herds [27]. This simultaneous occurrence can lead to recombination by template switching of the RNA polymerase. One of the breakpoints for recombination is in close vicinity to the L^pro^ coding region [29]. This is not of great concern, since Uddowla et al. [30] have shown that a chimeric FMDV with a BRBV L^pro^ is still attenuated.

## Conclusion

The BSL4vet conditions under which FMD infection trials have to be conducted limit the number, age and size of animals used and the statistical significance that can be obtained. Based on our results alone, it cannot be ruled out that leaderless virus is able to establish persistence in a small proportion of exposed animals. Nevertheless, our results demonstrate the essential role of L^pro^ for a productive FMDV infection. Without a functioning Leader proteinase, in our hands FMDV could neither establish an acute infection nor was it able to persist in the bovine nasopharynx. This strongly attenuated phenotype, which prevents clinical disease while retaining the full capsid-coding sequence, makes leaderless viruses a much safer alternative to wildtype FMDV for research, diagnostics (e.g. neutralization tests) and production of inactivated vaccines at lower biosafety levels.

## Supplementary Material

Figure_S1

Table_S1_Tissue_distribution

## Appendix

**Figure S1**. RNA *in situ* hybridization with the tongue of an acutely FMDV-infected heifer collected 2 dpi. (A–B) Treatment with the FMDV NSP 3D probe results in a high number of positive signals (=positive control), (B) shows a magnification of (A). (C–D) Incubation of a consecutive section of (A) with the negative control probe DapB shows no signal (=technical control), (D) shows a magnification of (C).

**Supplemental Table S1.** Numbers in the table represent log10 FMDV viral genome copies per mg of tissue collected at necropsy either at 24 hpi or 36 dpi in the WT group. Underlined numbers indicate positive virus isolation. neg, negative RT qPCR; –, sample was not collected; DSP, dorsal soft palate; DNP, dorsal nasopharynx; VSP, ventral soft palate; PHT, pharyngeal tonsil; LYX, epithelium of the larynx; lung 1/4, proximal cranial lobe; lung 2/4, distal cranial lobe; lung 3/4, distal mid lobe; lung 4/4, distal caudal lobe; RPLN, medial retropharyngeal lymph node; SMLN, submandibular lymph node; HLN, hilar lymph node.

**Table:**

Euthanasia	24 hpi		36 dpi					
Animal	755	769	758	773	753	764	768	770
Carrier			Carrier	Carrier				
DSP 1/5	neg	neg	2.4	3.2	neg	neg	neg	2.6
DSP 2/5	2.7	neg	neg	neg	neg	neg	2.9	neg
DSP 3/5	neg	neg	neg	2.0	neg	neg	2.9	neg
DSP 4/5	3.5	neg	neg	neg	neg	neg	neg	2.5
DSP 5/5	neg	neg	2.3	neg	neg	neg	neg	neg
DNP 1/5	neg	1.9	neg	2.7	neg	neg	2.7	3.8
DNP 2/5	neg	neg	neg	neg	neg	neg	2.8	3.7
DNP 3/5	neg	neg	3.5	neg	neg	2.7	neg	neg
DNP 4/5	neg	neg	3.9	neg	neg	neg	neg	neg
DNP 5/5	neg	neg	2.7	neg	neg	neg	neg	neg
VSP	neg	neg	neg	2.4	neg	neg	neg	neg
PHT	4.7	neg	3.5	2.5	neg	neg	3.1	neg
LYX	1.7	neg	2.4	2.4	2.4	neg	neg	neg
Lung 1/4	neg	neg	–	–	–	–	–	–
Lung 2/4	neg	neg	–	–	–	–	–	–
Lung 3/4	6.1	neg	–	–	–	–	–	–
Lung 4/4	1.9	neg	–	–	–	–	–	–
RPLN	1.3	1.9	4.2	3.2		3.4	3.3	
SMLN	neg	neg	4.9	4.5	5.1	3.8	6.1	5.5
HLN	neg	neg	–	–	–	–	–	–
