# Supplementary material for: Leaderless foot-and-mouth disease virus serotype O did not cause clinical disease and failed to establish a persistent infection in cattle
Source: Emerg Microbes Infect. 2024 Apr 29;13(1):2348526. doi: 10.1080/22221751.2024.2348526 (PMC11100440; doi:10.1080/22221751.2024.2348526)
Supplement: Figure_S1 [file TEMI_A_2348526_SM6922.pdf]

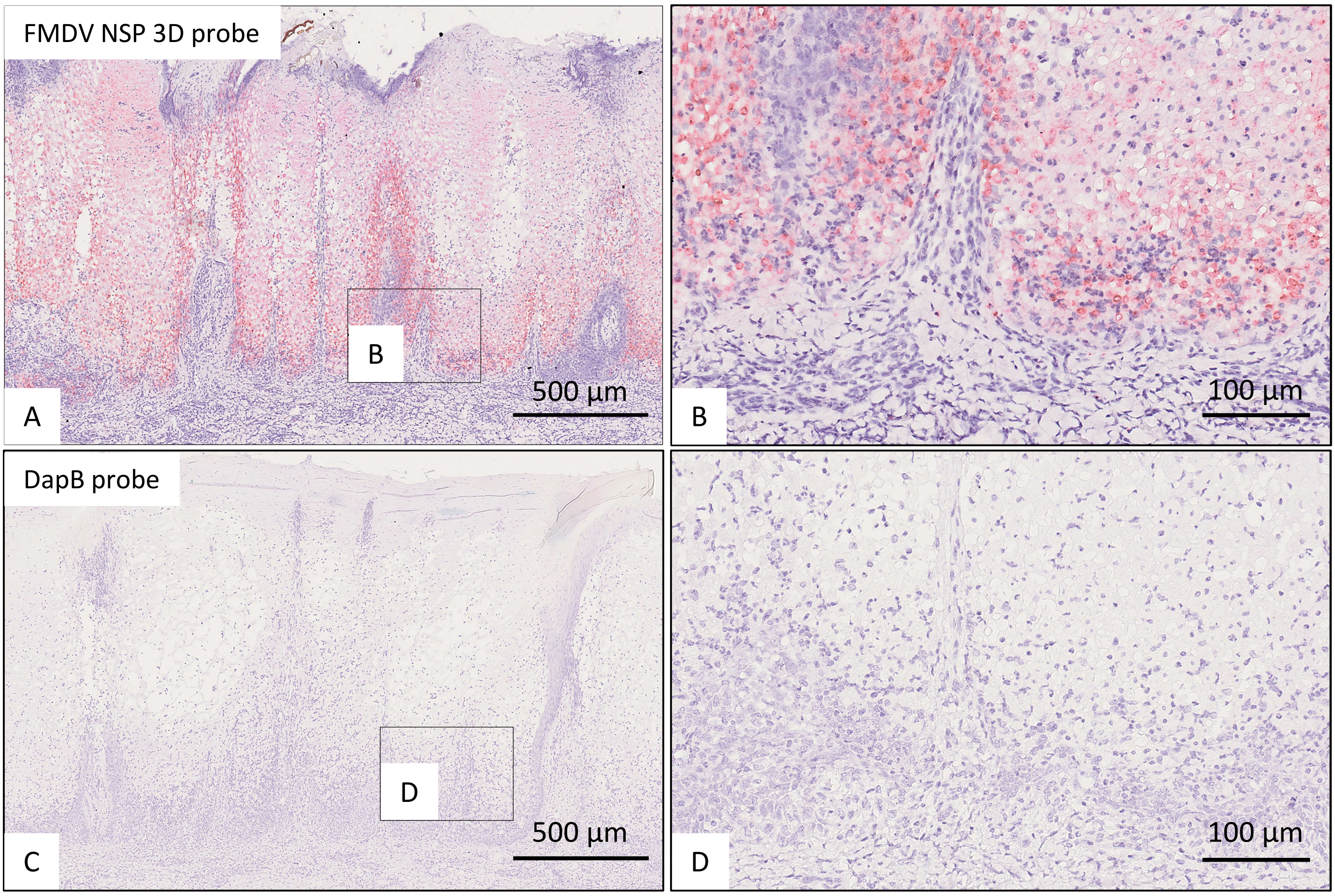

Suppl. Fig. RNA in-situ hybridization with the tongue of an acutely FMDV-infected heifer collected 2 dpi. A-B) Treatment with the FMDV NSP 3D probe results in a high number of positive signals (=positive control), (B) shows a magnification of (A). C-D) Incubation of a consecutive section of (A) with the negative control probe DapB shows no signal (=technical control), (D) shows a magnification of (C).
